# Temporal–Spatial Evolution and Influencing Factors of Coordinated Development of the Population, Resources, Economy and Environment (PREE) System: Evidence from 31 Provinces in China

**DOI:** 10.3390/ijerph182413049

**Published:** 2021-12-10

**Authors:** Junjie Cao, Yao Zhang, Taoyuan Wei, Hui Sun

**Affiliations:** 1 School of Economics, Shandong University of Technology, Zibo 255012, China; caojunjie@sdut.edu.cn (J.C.); 20517031029@stumail.sdut.edu.cn (H.S.); 2 CICERO Center for international Climate Research, 0318 Oslo, Norway

**Keywords:** sustainable development, PREE system, temporal–spatial evolution, polarization, factor analysis

## Abstract

Facing the increasingly severe friction among the domains of population, resources, economy and environment (PREE) in a system, theoretical guidance for the sustainable development of a PREE system can be obtained by exploring the coordinated development of a PREE system during its temporal–spatial evolution process. Based on the PREE data of 31 provinces in China from 2010 to 2019, this study uses a spatial measurement method to analyze the temporal and spatial evolution characteristics of the PREE systems of China’s provinces. The results show that the overall coordination level of China’s provincial PREE systems fluctuated but improved from moderate imbalance to moderate coordination. However, the differences in the regional coordination level first decreased and then increased. The distribution characteristics of the system coordination level changed from “high in the east and low in the west” to “high in the west and low in the east”, resulting in the “inversion” phenomenon of the system coordination level. The spatial correlation of the coordination level of the PREE system among provinces and cities gradually increased. The coordination level of the PREE system in the eastern, central and western regions was noticeably different, accompanied by different degrees of polarization and showing different dynamic evolution trends. In the analysis of influencing factors, it was found that seven factors, such as per capita GDP, the proportion of environmental pollution control investment to GDP and per capita energy production, promoted the coordinated development of China’s PREE system to varying degrees. The coordinated and stable development of China’s PREE system should be adjusted and optimized from the perspectives of different regions, scales and systems.

## 1. Introduction

As the main body of social development, the scale, structure and quality of the population, as well as the pursuit of a better life, consistently affect the level of economic development and the speed of social progress. Resources and the environment are not only the material basis and conditions for human survival and economic activities, but also the prerequisite and guarantee for sustainable social development. The central issue of sustainable development of human society can be summarized as the coordinated development of four subsystems: population, resources, economy and environment (PREE) [1]. At present, the demographic dividend in some countries such as China is declining due to population aging and the natural environment is being degraded due to the coal-based energy consumption structure and inefficient energy utilization rate, which are still the main contradictions restricting a high-quality development of China’s economy. The development plan of “advancing green development and promoting harmonious coexistence between people and resources”, proposed in China’s 14th Five-Year Plan, aims to alleviate the internal PREE friction to achieve sustainable development through the green transformation of the development mode [2]. The essence of sustainable development is the orderly, stable and coordinated development of the economy and society under the three constraints of population, resources and environment within a certain period of time under the conditions of science and technology.

Therefore, it is necessary to accurately analyze the relationship among and internal constraints of the PREE elements and establish a framework of system indexes to quantitatively evaluate and analyze the level of and possibility to accomplish sustainable development of a PREE system, with another aim being providing policy guidance for stimulating the potential of significant sustainable development in the region.

The coordinated development of a PREE system is a key element for China to achieve sustainable development. Studying the temporal and spatial evolution of the coordinated development of a PREE system and its influencing factors are conducive to summarizing and discovering the contradictions and problems existing in the process of regional sustainable development and formulating policies and guidelines for regional sustainable development. In addition, it can provide new ideas for how to improve sustainable development to achieve high-quality development in China.

Based on the review of the existing literature (see Section 2), we found that current research on PREE systems mostly focuses on theoretical mechanism analysis; few studies have explored the construction and measurement of a PREE system index framework, the comparison of coordinated development of a PREE system in different regions, or the factors affecting the coordinated development of a PREE system. Therefore, this study constructs a comprehensive and objective PREE system index framework. Taking into account the coordinated spatial interaction effects of regional systems, the spatial measurement analysis method and spatial error model are then used to analyze the temporal and spatial evolution characteristics of the coordinated development of the PREE system in different provinces of China and further explore its influencing factors. Therefore, our study provides a new scientific reference for the sustainable development of the PREE system in China.

The coupling coordination degree model can effectively analyze the coupling and coordination relationship among the four subsystems of PREE; the spatial measurement analysis method can clearly explore the spatiotemporal evolution characteristics of the coordinated development of the PREE system in different regions, while the spatial error model (SEM) can analyze the influencing factors of the coordinated development of a PREE system more accurately. To this end, this study’s research goals are as follows: (1) exploring the coordinated development mechanism of a PREE system and construct an index system; (2) revealing the spatiotemporal evolution characteristics of the coordinated development of a PREE system; (3) identifying the affecting factors of the coordinated development of a PREE system; (4) providing reference for formulating sustainable development policies for regional PREE systems.

## 2. Literature Review

As early as the 1950s–1960s, against a backdrop of increasingly depleted resources and deteriorating environment, people began to think about how to realize rapid economic and social development and, at the same time, rationally utilize resources and protect the environment. Sustainable development was first mentioned in the World Conservation Strategy of the International Union for Conservation of Nature in 1980 and the concept of sustainable development was defined in the report Our Common Future, published in 1987 [3]; the report aroused, in scholars from all over the world, the desire to evaluate the concept of sustainable development and actively explore the coordinated development of different subsystems aimed to realize collective sustainable development. For example, Shmelev et al. [4] proposed a comparative sustainability assessment method based on environmental expansion input–output analysis and multi-criteria decision-making assistance to discuss the macro-sustainability of various countries. Patterson et al. [5] used the ecological footprint method to explore the sustainable development of the coupling and coordination of regional tourism and ecological environment. Nilashi et al. [6], for the first time, applied the technology of fuzzy clustering and supervised machine learning to the evaluation of national sustainability to expand the sustainability assessment system.

At present, China’s research trend in terms of coordinated development among different systems is generally consistent with the international research trend, which is mainly directed at the measurement of the coupling of coordination and sustainable development of binary and ternary systems (3E). The coupling of a binary system refers to the relationship between economy and energy, energy and environment, and environment and economy. Relatively to the binary relationship between economy and energy, scholars all over the world think that these two domains have a stable two-way causal relationship that varies significantly among different regions [7,8,9]. Regarding the relationship between energy and environment, some scholars believe that there are long-term and short-term differences in the influence of different energy consumption amounts and structures on environmental pollution, which can be effectively reduced by improving energy utilization efficiency [10,11]. Finally, the relationship between environment and economy is discussed on the basis of the environmental Kuznets curve (EKC), which has always been controversial due to its limitations and endogenous defects, according to some scholars. The relationship between economy and environment has various forms; economic growth and environment are interactive systems, whereby environmental deterioration affects economic growth and income improvement. However, the EKC does not permit to establish an endogenous model to discuss the relationship between these two domains [12], although some supporters use data from different countries and regions to prove that there is an “inverted U-shaped” relationship among income level, economic growth and environmental pollution [13,14].

Concerning the 3E (energy, economy and environment) system, which constitutes another important research focus in the field of sustainable environment, at present, three main aspects have been investigated. Firstly, the relationship between the coordination degree measurement method and the system, which is studied with various models that measure the coordination degree, such as the coupling coordination degree model, the PLSPM-GIA model, etc. [15], or econometric models that analyze the interactions among energy, economy and environment [16,17]. Secondly, researchers have been analyzing the coordinated development of the 3E system in a certain region, i.e., a single province or a specific region, in a certain period of time, with the aim of proposing some governance suggestions according to the problems existing in the coordination process of the 3E system in the studied region [18,19]. Thirdly, the spatial correlation and dynamic evolution of a 3E system has been evaluated from the perspective of geography; in this sense, researchers have investigated the geographical spatial differentiation of the coordination level of a 3E system by using spatial measurement tools, as well as estimating and analyzing the dynamic evolution of the coordination level of a 3E system [20,21].

Finally, as an extension of the 3E system, some scholars have proposed the concept of the 4E system, which comprises the energy, economy, environment and ecology domains; the assumption is that each system has the attributes of game and synergy under the goal of ecological civilization construction, making the coordinated development of a 4E system crucial for ecological civilization construction [22]. Some scholars have improved upon the 3E system and proposed the PREE system, which comprises the domains of population, resources, economy and environment, with economy as the core and the sustained, stable and orderly coordinated development of an economic system under the constraints of population, resources and environment being the essence of the coordination of a PREE system [23].

In summary, current research on the PREE system is mostly based on theoretical mechanism analysis, which lacks the construction and measurement of a PREE system index framework, the comparison of coordinated development of the PREE system in different regions and the inquiry into the factors affecting the coordinated development of a PREE system. Therefore, this thesis attempts to contribute to research on PREE systems in these aspects.

## 3. Analysis of Coordinated Development Mechanism of PREE System

**Figure 1:**
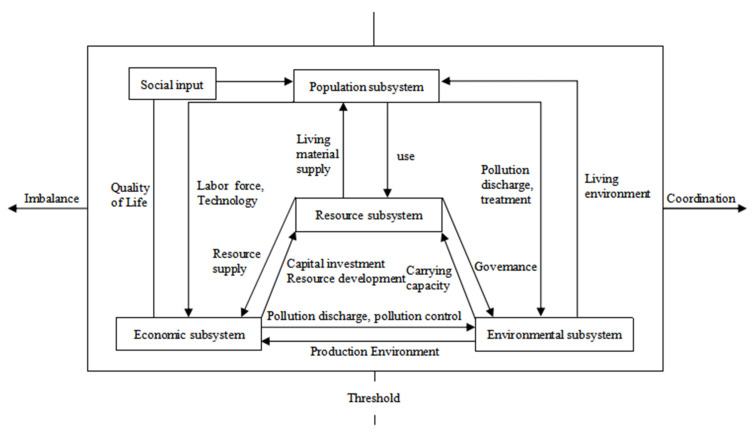
PREE system operating mechanism structure diagram. Source: modified based on Figure 1 in Zeng et al. [1].

Population, resources, economy and environment are interdependent and interrelated domains that form a complex system, called a population–resources–economy–environment system. Sustainable development of a PREE system is mainly reflected in the rationality of the internal structure of the system, as well as the coordination and effectiveness of the interaction mechanism among subsystems and the coordinated development within each subsystem, with the degree of coordination among subsystems being the main evaluation criteria. Combined with the concept of sustainable development, under the basic structure of the coordinated development of population, resources, environment and economy proposed by Zeng et al. [1], the coordinated development mechanism among systems is here reinterpreted. The system structure is shown in Figure 1.

In a PREE system, the population subsystem is the main body and the term “people-oriented” is the core concept of scientific development. People-oriented means taking people’s survival as the foundation—everything is aimed at people’s well-being. Chen [24] argues that, by sending labor and investment to the resource development industry, employment pressure can be effectively resolved and the development and utilization of resources can be promoted. In the system, the population subsystem promotes the improvement of the economic level, resource availability and environmental quality by providing population (labor), science and technology to the other three subsystems; in turn, these provide the population subsystem with better quality of life, living materials and living environment. However, the disharmony among population size, structure and quality brings employment pressure, consumption pressure, waste of resources and environmental pollution [25], which not only restricts economic development, but also brings great pressure to the resources and environmental subsystems. It can be seen that a PREE system is a closed-loop system with the population subsystem constituting its main body and the fundamental purpose of the coordinated operation of the other three subsystems being meeting the overall requirements of the population subsystem.

The resource subsystem is the material basis of the PREE system and the social and economic development is the result of the joint action of human and natural resources. Economic development promotes the progress of science and technology, which, in turn, promotes the improvement of the resource utilization rate and environmental transformation ability. Meanwhile, it also expands the connotation and boundary of natural resources and increases the resource stock, while the improvement of environmental quality not only improves the stock level and quality level of resources, but also increases the supporting capacity of the resource system. Wei et al. [23] argue that there is a mutual restriction between the increase in resource stock, due to economic and social development promoting technological progress, and the consumption of resources by the population and economic subsystems. When one party changes greatly, it breaks the original balance of the system. The coordinated development of the resource subsystem should consider the carrying capacity of the resource environment in the region.

The economic subsystem is the core of the PREE system. During its operation, the economic subsystem should not only meet the material and capital investment for other subsystems, but also meet its own productive investment. When the material and capital investment of other systems is met, it reduces the capital investment in its own production and inhibits economic growth. However, when investing in other subsystems, other systems adjust the economic structure, improve the economic benefits and indirectly promote economic growth by adjusting the quality of elements such as human resources, natural resources, production environment, etc. Xiao et al. [26] argue that, by adjusting the economic development structure and improving economic efficiency, CO_2_ emissions can be effectively reduced; further, they believe that, for the operation of China’s economic subsystems at the present stage, not only we should pay attention to the trend of economic growth, but we should also pursue higher economic benefits and a better economic structure, so as to achieve high-quality economic development and sustainable development with various systems.

The environmental subsystem is the spatial support of the PREE system as it provides spatial support for all the activities in the system. There are two types of relationships between environmental carrying capacity and social development, namely, coordination and conflict. When the economic development level is on the left side of the inflection point of the environmental Kuznets curve, with the economic development and the increase in human activities, the social costs of environmental protection are low, leading to the aggravation of environmental pollution and the decline of the environmental carrying capacity. On the other hand, when the economy develops to a certain extent, the social costs caused by environmental pollution are higher than other social costs, which prompts the government and individuals to provide the necessary financial and human support for environmental improvement. Mirza et al. [27] argue that energy consumption and economic growth have increased environmental pollution and that improving energy efficiency and reducing the use of fossil fuels are effective measures to reverse regional environmental degradation. Meanwhile, as the environment is the carrier of resources, the improvement of environmental quality effectively improves the stock and quality of resources, promotes economic development and meets people’s living needs; on the contrary, the deterioration of environmental quality restricts economic development by reducing the stock and quality level of resources.

To sum up, the coordination of and conflict among different subsystems exist in the operation mechanism of a PREE system, which reflects the internal factors of sustainable development. The closed-loop structure of the subsystems forms a complex closed-loop system. When the influence of external control factors on the internal system state reaches a threshold, the subsystem operation state in a PREE system changes from a disordered state to a macro-orderly state and the system achieves coordination.

## 4. Research Design

### 4.1. Data Sources and Construction of Index System

#### 4.1.1. Data Sources

In this study, we used the data of 31 provinces, municipalities and autonomous regions in China from 2010 to 2019. We extracted the original data of all the indicators from the following: China Statistical Yearbook from 2011 to 2020, China Statistical Yearbook on Environment from 2011 to 2020 and China Energy Statistical Yearbook from 2011 to 2020; among them, some indicators for Xinjiang, Tibet and Hebei are missing for the year 2019 and interpolation was used to supplement them.

#### 4.1.2. Construction of Index System

Index selection is the basis for the coordinated development of China’s provincial PREE system. Whether the index selection is reasonable or not, it directly affects the scientificity and representativeness of the evaluation results. Based on the existing research results, this paper selected 29 indexes according to the principles of data availability and scientificity and constructed an index system for the coordinated development of China’s PREE system, as shown in Table 1.

In the index system for coordinated development, the coordinated operation of the population subsystem is mainly reflected in the coordinated development of scale, structure and quality [28]; therefore, for this paper we selected six indexes, such as population quantity and proportion of urban population, to measure the population subsystem. For the economic subsystem, the economic development level, operation structure and output efficiency need to be considered [29] and six indexes, such as per capita GDP and input–output ratio, were selected to reflect it. The resource subsystem needs to be analyzed under the two aspects of resource stock and utilization [30]; therefore, seven indexes, such as per capita water resources and water consumption per CNY 10 thousand GDP, were selected for measurement. The environmental subsystem is considered from three aspects, namely, environmental pollution, environmental treatment and environmental protection construction [31,32]; therefore, we selected ten indexes, such as urban wastewater discharge and the proportion of environmental pollution control investment to GDP. Among them, the larger the index value is, the more beneficial to the coordinated development of system, meaning the index is positive, while the opposite means the index is negative. Population density, per capita investment in fixed assets, per capita retail sales of consumer goods and per capita water resources were obtained by original data calculation.

**Table 1:** Index system of coordinated development of population–economy–resources–environment. Data source: China Statistical Yearbook, China Environmental Statistical Yearbook, China Energy Statistical Yearbook.

Target Layer	Subsystem Layer	Criterion Layer	Index Layer (Unit)	Index Direction	References
PREE system coordinated development index	Population subsystem	Population size	Population quantity (person)	—	[22,23,29,31]
			Population density (person/km^2^)	—	[29,30,31]
		Population structure	Aging coefficient (%)	—	[22,23,31]
			Male/female proportion (%)	—	[29,30,31]
			Proportion of urban population (%)	+	[22,23,29,30,31]
		Population quality	Population with college degree or above (ten thousand people)	+	[23,28,29,30]
	Economic subsystem	Economic level	Per capita GDP (CNY/person)	+	[15,17,18,19]
			Investment in fixed assets per capita (CNY/person)	+	[15,19,30,31]
			Retail sales of social consumption goods per capita (CNY/person)	+	[17,18,19,31]
		Economic structure	Proportion of primary industry GDP to total GDP (%)	—	[17,18,19,20,30,31]
			Proportion of tertiary industry GDP to total GDP (%)	+	[17,18,19,20,30,31]
		Economic efficiency	Input–output ratio (%)	+	[15,18,19,20,31]
	Resource subsystem	Resource conditions	Per capita water resources (^3^/person)	+	[18,19,20,30]
			Cultivated land area (thousand hectares)	+	[19,20,31]
			Per capita energy production (ton coal equivalent/person)	+	[17,18,19,20,30,31]
			Forest area (ten thousand hectares)	+	[23,29,30,31]
		Resource utilization	Water consumption per CNY ten thousand GDP (m^3^/CNY ten thousand)	—	[18,19,20,23,29,30]
			Energy consumption per CNY ten thousand GDP (ton coal equivalent/CNY ten thousand)	—	[15,18,19,20,23,29,30]
			Grain output per unit (ton/ hectare)	+	[15,23,29,31]
	Environmental subsystem	Environmental pollution	Urban wastewater discharge (ten thousand cubic meters)	—	[18,19,20,23,29,30,31]
			Urban exhaust emissions (ten thousand tons)	—	[18,19,20,23,29,30,31]
			Emissions of industrial solid waste (ten thousand tons)	—	[18,19,20,23,29]
			Urban domestic garbage removal (ten thousand tons)	—	[18,19,20,23,30,31]
		Environmental governance	Urban sewage treatment rate (%)	+	[18,19,20,29,30]
			Utilization amount of industrial solid waste (ten thousand tons)	+	[18,19,20,23]
			Industrial waste gas treatment capacity (ten thousand cubic meters/hour)	+	[18,19,20,23,29,30,31]
			Harmless treatment rate of domestic garbage (%)	+	[18,19,20,23,29,31]
		Environmental protection construction	Forest coverage rate (%)	+	[20,30,31,32]
			Proportion of environmental pollution control investment to GDP (%)	+	[15,18,19,20,31]

### 4.2. Research Methods

To calculate the coupling coordination degree of a PREE system, we standardized the above indexes and calculated the index weights by the entropy method described in [33]. Then the evaluation level of each system is obtained by multiplying the standardized data with their weights and the coupling coordination level of PREE system is calculated by coupling coordination degree model [34]. Finally, a spatial econometric analysis method and a spatial error model are used to analyze the spatiotemporal evolution characteristics and influencing factors of the coupling coordination development of China’s provincial PREE system. The detailed methods are described as follows.

#### 4.2.1. Standardized Processing of Data

The indexes were standardized. The positive index formula is

(1) $$x_{ij}^{’}=\frac{x_{ij}-min\left(x_{ij}\right)}{max\left(x_{ij}\right)-min\left(x_{ij}\right)}$$

The negative index formula is

(2) $$x_{ij}^{’}=\frac{max\left(x_{ij}\right)-x_{ij}}{max\left(x_{ij}\right)-min\left(x_{ij}\right)}$$

#### 4.2.2. Calculating Weights by the Entropy Method

The entropy method was used to give weight to each index [31]. Firstly, the proportion *P_ij_* of the index value of the *i*th object under the *j*th index was calculated with the following formula:

(3) $$P_{ij}=\frac{x_{ij}}{\sum_{j=1}^{m}x_{ij}}$$

Secondly, the information entropy *E_j_* was calculated with the formula

(4) $$E_{j}=-\frac{1}{\ln\left(m\right)}\overset{m}{\underset{i=1}{\sum }}P_{ij}\ln P_{ij},\;\;i=1,2,3,\cdots ,m;j=1,2,3\cdots ,n$$

where if $P_{ij}=0$, then $P_{ij}\ln P_{ij}$ is replaced by $\underset{P_{ij}\to 0}{\lim}P_{ij}\ln P_{\mathrm{ij}}=0$.

Finally, the weight *W_j_* of the index was calculated with the formula

(5) $$W_{j}=\frac{1-E_{j}}{\sum_{j=1}^{n}1-E_{j}}(0\;\leq \;W_{j}\leq \;1)$$

#### 4.2.3. Subsystem Evaluation Model

The evaluation index of each subsystem was calculated with the formula

(6) $$F_{j}=\sum_{j=1}^{m}W_{j}x_{ij}^{’}\;(j=1,\;2,\;3,\;4)\;$$

where *W_j_* is the weight of each index, $x_{ij}^{’}$ is the standardized value of each index and *F_j_* is the evaluation value of the subsystem.

#### 4.2.4. Coupling Coordination Degree Model

The PREE system coupling degree was calculated with the formula

(7) $$C={\left\{\frac{F_{1}\times F_{2}\times F_{3}\times F_{4}}{{\left[\left(F_{1}+F_{2}+F_{3}+F_{4}\right)/4\right]}^{4}}\right\}}^{\frac{1}{4}}$$

(8) $$T=\alpha_{1}F_{1}+\alpha_{2}F_{2}+\alpha_{3}F_{3}+\alpha_{4}F_{4}$$

(9) $$D=\sqrt{CT}$$

where *C* represents the coupling degree, *T* is the coordination index of a PREE system and α_1_, α_2_, α_3_ and α_4_ are the undetermined weights of four systems. In the evaluation process, it was considered that the four systems had the same importance—α_1_ = α_2_ = α_3_ = α_4_ = 0.25—and D is the coupling coordination degree. The coupling coordination degree of a PREE system is divided into 10 types [35], as shown in Table 2.

#### 4.2.5. Spatial Autocorrelation

The spatial autocorrelation test was used to test the spatial correlation of the PREE system coupling coordination degree for each province. The formula used is

(10) $$I=\frac{n\sum_{i=1}^{n}\sum_{i=1}^{n}W_{ij}\left(x_{i}-\bar{x}\right)\left(x_{j}-\bar{x}\right)}{\sum_{i=1}^{n}\sum_{j=1}^{n}W_{IJ}{\left(x_{i}-\bar{x}\right)}^{2}}$$

where $\overset{-}{x}$ = $\frac{1}{n}\sum_{i=1}^{n}x_{i}$, n is the total number of regional units in the research area, *x_i_* the variable value of regional unit I and *W_ij_* is the spatial weight matrix, The adjacent ones between provinces and cities are marked as 1, and the non-adjacent ones are marked as 0.

The local Moran index was used to measure the correlation of the coordination level of the PREE systems among the provinces in China.

(11) $$I_{i}=\frac{\left(x_{i}-\bar{x}\right)\sum_{j=1}^{n}W_{ij}\left(x_{j}-\bar{x}\right)}{S^{2}}$$

where *S* is the standard deviation, *x_i_* is the variable value of regional unit *i*, *x_j_* is the variable value of regional unit *j* and *W_ij_* is the spatial weight matrix.

#### 4.2.6. Kernel Density Function

The advantage of the kernel density function is that it avoids the subjectivity of setting the estimation form of the parameter model. The distribution characteristics of the numerical value were studied from the data themselves with the formula

(12) $$f_{n}\left(x\right)=\frac{1}{nh}\overset{n}{\underset{i=1}{\sum }}K\left|\frac{x_{i}-x}{h}\right|$$

where *n* is the number of observations, *x* is the mean value, *h* is the smoothing parameter and *K* is the kernel function.

#### 4.2.7. Spatial Error Model

Spatial error model is mainly used when there is spatial autocorrelation among residual terms. The formula is

(13) $$Y=\beta_{I}X_{it}+\xi_{t}+\sigma_{i}+\varepsilon_{it},\;\varepsilon ={\lambda W\varepsilon }_{it}+\mu_{it}$$

where *ξ_t_* and σ*_i_* represent the time and space effects, *λ* is the spatial correlation error coefficient, *μ_it_* is the random error vector with normal distribution and *W* is the spatial weight matrix.

## 5. Temporal–Spatial Differentiation of Coordinated Development Level of China’s Provincial PREE System

### 5.1. Change Characteristics Analysis

#### 5.1.1. Overview of Coordinated Development Level

From 2010 to 2019, the coordinated development level of China’s PREE system fluctuated and rose from a slight imbalance to a moderate coordination. In the research period, 2010–2019, its change process can be divided into two stages. (1) In the high growth stage (2010~2013), the overall coordination level of China’s PREE system was low; during this stage, the 12th Five-Year Plan was also at the beginning of its implementation. The 12th Five-Year Plan aimed at “green development and building a resource-saving and environment-friendly society”, permitting China to develop its economy at a high speed; further, it also put forward new requirements for environmental governance and resource utilization. During this period, China strengthened environmental protection, enhanced resource conservation and management, promoted ecological protection and restoration, vigorously developed a circular economy and boosted the rapid improvement of the coordination level of its PREE system. At this stage, the eastern region’s average value of the coordinated level was higher than that of other regions, taking the lead in the country. (2) In the low growth stage (2014–2019), the PREE system achieved coordinated development and resource conservation, while intensive utilization and comprehensive environmental control were continuously implemented; nonetheless, the growth rate showed a downward trend. The level of coordinated development and the growth rate showed an inverted U-shaped linear relationship. With the continuous improvement of the coordination level, coordinated development no longer relied on simple means such as decreasing pollutant emissions and reducing resource waste; the influencing factors gradually changed from the coordination of a single subsystem to the coordination of multiple subsystems and the difficulty in improving the coordination level of the system gradually increased.

#### 5.1.2. Coordinated Development at the Region Level

**Figure 2:**
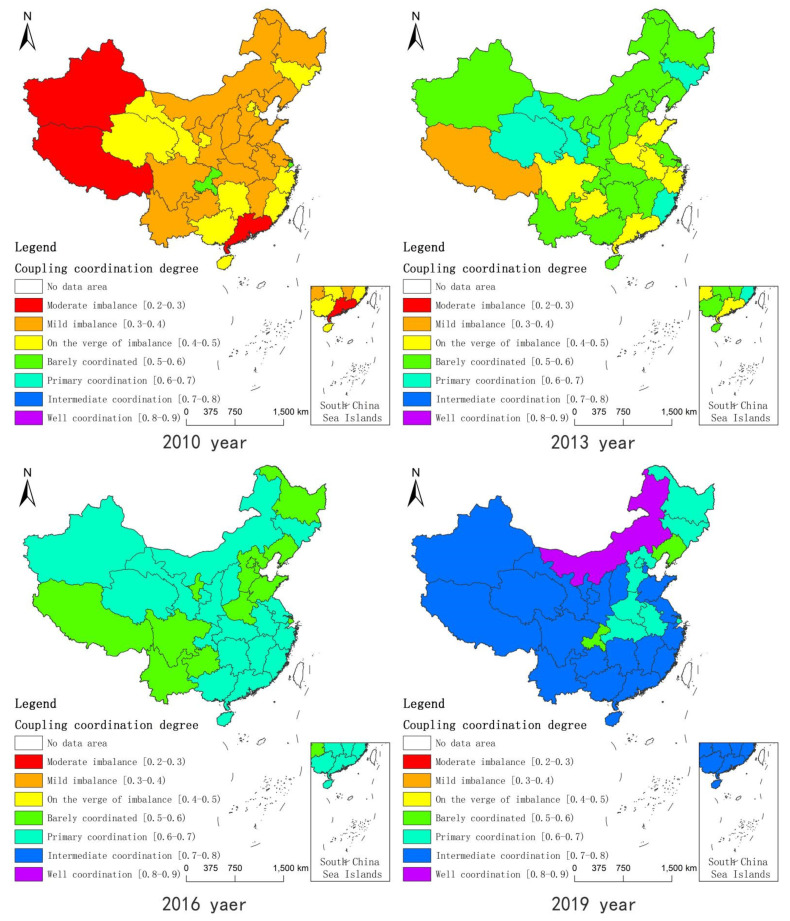
The spatial distribution of coordination state of various provinces and cities in China in 2010, 2013, 2016 and 2019. Source: map data obtained from the China National Geographic Information Center.

Selecting two-year intervals, the coordination degree of China’s PREE system was used as vector in the maps shown in Figure 1. In 2010, except Chongqing and Shanghai, all the other provinces and cities were in an imbalanced state, among which 10 provinces and cities were on the verge of imbalance, 16 provinces and cities were in mild imbalance and 3 provinces and cities were in moderate imbalance. By 2013, the imbalance had improved and eight provinces and cities were in imbalance; only the Tibet Autonomous Region was in mild imbalance and no provinces and cities were in moderate imbalance, most of which were on the verge of imbalance and barely coordinated. In 2016, there were no imbalanced provinces and cities and the overall country was in a state of coordination, with only two states of bare coordination and primary coordination; the difference in the coordination level among provinces and cities was the lowest. In 2019, there were still provinces and cities that were barely coordinated, with eight provinces and cities in a primary coordination state, most of which were in an intermediate coordination state, with only the Inner Mongolia Autonomous Region in a good coordination state. It can be seen that the coordinated development level of China’s provinces and cities was in a dynamic change process and, overall, it trended towards coordinated development; however, the regional differences first decreased and then increased. Combining Table 3 and Figure 2, it can be seen that the low-value areas of the PREE system gradually showed a better trend in the east and worse in the west, better in the north and worse in the south; further, the coordinated development level of the PREE system in the three northeastern provinces overall lagged behind the other areas. During the research period, the distribution characteristics of the system coordination level changed from “high in the east and low in the west” to “high in the west and low in the east”, resulting in the phenomenon of the “inversion” of the coordination level.

### 5.2. Spatial Change Analysis

#### Spatial Autocorrelation Analysis

**Figure 3:**
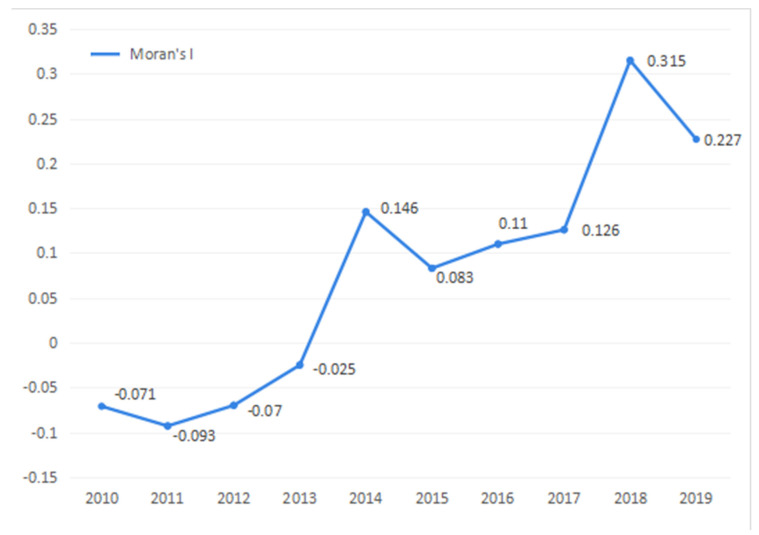
Changes in the overall Moran’s I index of the coordinated development level of China’s provincial PREE system. Source: own elaboration.

With the increasing popularity of applying spatial reference data sets to scientific applications, it is important to fully infer and accurately evaluate the spatial variation process of each variable. When spatial reference data are used, some variables are potentially interdependent among the observed data in a nearby distribution area. Hence, it is necessary to identify whether the interdependencies exist among various factors. Spatial autocorrelation can be used to describe the spatial interdependences and is divided into two categories: global type and local type. Moran’s I index [36] is used to detect both types by testing the distribution characteristics of variable factors and evaluate the limited sample properties of variables. Global Moran’s I index is used to detect the spatial dependence among variables as a whole and local Moran’s I index is used to detect the spatial dependence of local variables after passing the global Moran’s I index test. The GeoDa [37] software was used to calculate the global Moran’s I index of coordination level of China’s provincial PREE system and the Monte Carlo method in the software was used to test the significance of the global Moran’s I index. The results are shown in Figure 3. The spatial correlation of the coordination level of China’s provincial PREE system increased yearly. The Moran’s I index in 2014, 2017, 2018 and 2019 passed the significance test at the level of 10%, 5% and 1%, respectively. The coordination level of the PREE system in various provinces and cities in China in the years under analysis showed spatial correlation.

The local Moran’s I index was analyzed for the four years that passed the global Moran’s I index significance test. In the process of calculation, Hainan Province resulted not connected with other provinces. By modifying the spatial weight matrix, Hainan Island was connected with Guangdong Province and Guangxi Province to solve the “isolated island” problem. The LISA cluster results of 31 provinces and cities were counted, as shown in Table 4. From the results, it can be seen that the high–high clusters mostly appear in the western region after 2016, including Gansu Province and Ningxia Hui Autonomous Region, for three times; Xinjiang Uygur Autonomous Region, Qinghai Province and Tibet Autonomous Region, for two times; and Sichuan Province, for one time, which had high spatial stability. However, the low–low clusters mostly appeared in the eastern regions, including Shandong Province in 2014, Hebei Province and Tianjin City in 2017 and Liaoning Province in 2018. These clusters had a certain spatial mobility. Cluster results further verified the conclusion that the coordination level of China’s PREE system was “high in the west and low in the east”. Meanwhile, it is worth noting that, after 2016, the differences in the coordination level among provinces and cities increased yearly and the spatial correlation also increased yearly, which means that the differences in the coordination level changed from being differences among single provinces and cities to being regional differences.

### 5.3. Dynamic Evolution Analysis

**Figure 4:**
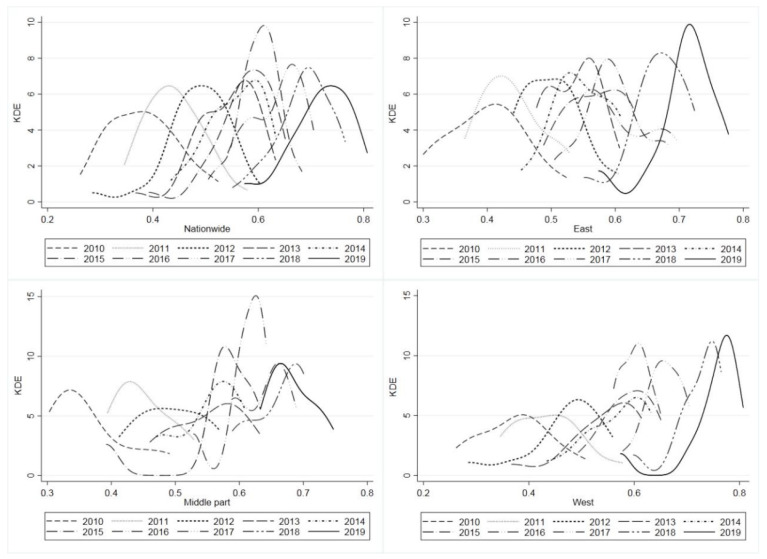
The dynamic evolution of nuclear density of system coordination level by region. Source: own elaboration.

The nonparametric kernel density estimation further revealed the absolute difference and dynamic evolution law of the coordination level of China’s provincial PREE systems. The Gaussian kernel function is superior to other kernel functions due to less grouping. Therefore, the Gaussian kernel function was used to estimate the kernel density of the overall system coordination level of 31 provinces in China and that of the eastern, central and western regions from 2010 to 2019; then, the kernel density distribution map was drawn. The dynamic evolution trend of the coordination level between the overall system and the PREE system in different regions was analyzed according to the kernel density curve, as shown in Figure 4.

In overall terms, the nuclear density center of the coordination level of the PREE system visibly moves to the right from 2010 to 2019, with a large movement range in the early stage and a smaller one in the latest stage. The peak height reaches the highest value in 2016; then, it decreases yearly. The wave width first narrows and then widens, changing from a wide peak type to a sharp peak type and then again to wide peak type. The peak tail changes from right-tail to left-tail and its thickness increases. The nuclear density curves for 2013, 2014 and 2017 all show weak “double peaks”. The change in the nuclear density curve from 2010 to 2019 shows that the overall coordination level of the PREE system increased yearly, the difference in the coordination level among regions first decreased and then increased, the proportion of low-value areas of coordination level increased and the phenomenon of “polarization” was severe in individual years, with a trend of two different polarizations.

In terms of different regions, the movement trend of nuclear density center in the coordination level of the PREE system in three regions is roughly the same as the overall one, but there are differences in the waveform. Among them, the eastern region’s bimodal pattern in 2013 is similar to the overall one, but the bimodal pattern in 2017 appears on the right side, which is opposite to the overall one. In 2012 and 2016, there is a long right tail; in 2019, the peak height shows the highest value and the peak width is reduced, changing from a wide peak type to a sharp peak type, while the left-tail is longer and the thickness decreases, with a tendency to evolve into double peaks. This shows that, during the research period, the difference in the coordination level among provinces and cities in the eastern region decreased to the minimum in 2019 and the proportion of low-value areas of the coordination level in the eastern region decreased, but the phenomenon of two different polarizations was more significant.

The change in nuclear density of the system coordination level in the central region is chaotic, with the highest peak in 2016 and then a yearly decrease, which indicates that the overall peak height in 2016 is greatly affected by the central region and the difference in the coordination level among regions is first decreased and then increased. The peak tail changes from right-tail to left-tail and then again to right-tail. In 2015, the left-tail is the longest; then, the tail shortens and rises. In 2019, it showed a double-peak trend. The proportion of high-value areas of regional coordination level increased and the regional system coordination level further increased, accompanied by the polarization phenomenon. The western region generally shows a long left-tail—except in 2010, 2011 and 2016. In 2018 and 2019, the left-tail is longer and higher and it changes from a single peak to a weak double peak. The peak width in the region decreases and changes from a wide peak type to a sharp peak type and the peak height shows an upward trend. From 2016 to 2019, the peak height in the western region is higher than that in 2016.

The regional differences in western China were mainly characterized by “high–low” differences. The proportion of low-value areas in the region gradually increased, the difference in the regional coordination level was smaller than the overall difference and the phenomenon of two different polarizations was prominent. It can be seen that the dynamic evolution of the coordination level of China’s PREE system in different regions and different periods is the result of interaction and symbiosis between regional characteristics and period characteristics.

## 6. Analysis of Influencing Factors of Coordinated Development Level of China’s Provincial PREE System

### 6.1. Factor Selection and Model Construction

The coordination level of China’s provincial PREE system is affected by many factors. Based on existing research works, after statistically studying the relevant literature on factors affecting sustainable development, combined with the development characteristics of each subsystem, we analyzed seven aspects: population structure [38], population quality, resource utilization [39], resource conditions [40], economic level, economic structure [41] and environmental protection construction [42]. To perform the analyses, we considered seven explanatory variables: per capita GDP (GDP), proportion of tertiary industry GDP to total GDP (TI), proportion of environmental pollution control investment to total GDP (EP), forest coverage rate (FC), per capita energy production (EPC), grain output per unit (GY), proportion of urban population (UP) and population with college degree or above (PC).Variable descriptive statistics are shown in Table 5. We took the logarithm of each explanatory variable to generate the corresponding ln x variable and perform the regression analysis.

It has been proved above that the coordination levels of PREE systems have spatial correlations, which indicates that a spatial econometric model is better than other types of models in explaining the roles of the influencing factors [43]. However, the choice of spatial econometric model shall not be limited to the spatial lag model (SLM) or the spatial error model (SEM), but shall also consider the spatial Dubin model (SDM) to explain the direct and indirect effects of influencing factors [44]. As an extension of the spatial error model (SDM), the spatial Durbin error model (SDEM) contains the spatially lagging explanatory variables, which can measure the exogenous interaction between explanatory variables and error terms and better explain the spillover effect of variable factors [45]. Which model to use needs to be tested. The LM test [44] was carried out for the model and the test results are shown in Table 6. The test results show that the LM-Error was significant at 1%, the LM-Lag was not significant and the RobustLM-Error and RobustLM-Lag were both significant at 1%. Therefore, the SEM model is chosen to better explain the roles of the influencing factors.

The error items in the SEM model may include the individual-fixed effect and individual-random effect; therefore, the Hausman test was selected to determine the specific effect type and the test results are shown in Table 7. From the test results, it can be seen that the *p*-value was significant and the original hypothesis was rejected, so the fixed effect model was selected.

### 6.2. Results Analysis

The time-fixed effect and individual-fixed effect were regressed by SEM and the regression results are shown in Table 8. According to the comparison between R^2^ and Log-likelihood, the individual-fixed effect was clearly superior to the time-fixed effect; therefore, SEM with individual-fixed effect was selected to analyze the results.

The coupling coordination degree value (Y) of the sample data ranges from 0.26 to 0.81, and its fitted value ranges from 0.39 to 0.78, all of which are within a reasonable range. The regression results have theoretical significance. The results show that, except for the factors affecting the number of people with college education or above, the other seven factors were all significant at the level of 1% significance, indicating that they all have a positive influence on the coordinated development of PREE system. Among them, the coefficient of per capita GDP is 0.3968 and the coefficient of tertiary industry GDP to total GDP is 0.2708. With the expansion of the economic scale and the optimization of the economic structure, the positive promotion influence of two factors on the coordinated development of the PREE system becomes more and more obvious, which shows that the operation of China’s economic subsystem at present should not only focus on the quantity of economic growth, but also pursue higher economic benefits and a better economic structure. The coefficient of proportion of environmental pollution control investment to GDP is 0.0343 and the coefficient of forest coverage rate is 0.2460, which shows that the reduction in environmental pressure and the improvement of environmental control ability can provide better space support for the coordinated development of the PREE system and play a positive role in promoting the sustainable development of the PREE system. The coefficient of energy production per capita is 0.1427 and the coefficient of grain output per unit is 0.1692. The resource subsystem is the material basis for the coordinated operation of the PREE system. The coordinated development level of the system can be improved by improving the resource stock and resource utilization rate. The coefficient of proportion of urban population is 0.7066, which shows that the level of social development improves constantly, as are the social infrastructure and people’s living standards, thus continuously promoting the coordination level of the PREE system. At present, China faces the problem of the declining demographic dividend. In the case in which the population scale is difficult to change, the coordination and stability of the population subsystem should be carried out under two aspects, namely, improving the population quality and improving the population structure.

## 7. Conclusions and Policy Implications

### 7.1. Conclusions

Based on the panel data of 31 provincial-level administrative regions in China from 2010 to 2019, this study calculated the coordination level of PREE systems at the provincial level in China and analyzed the temporal–spatial evolution characteristics and influencing factors of the system coordination level.

Our study found that there were temporal–spatial differences in the coordination level of China’s provincial PREE system in the research period and the overall coordination level showed fluctuations and improvements. During the research period, the coordination level can be mainly divided into two stages, high growth (2010~2013) and low growth (2014~2019); the difference in the coordination level among provinces first decreased and then increased. The distribution characteristics of the system coordination level changed from “high in the east and low in the west” to “high in the west and low in the east”, resulting in the “inversion” phenomenon of the coordination level. The spatial correlation of the coordination level of the PREE system among provinces gradually increased. High–high clusters were mostly in the western region with high spatial stability, while low–low clusters were mostly in the eastern region with a certain spatial mobility. With the strengthening of inter-regional cooperation, the difference in the system coordination level changed from single provincial and municipal differences to regional differences.

During the research period, the overall coordination level of the PREE system increased yearly. The difference in the coordination level among regions decreased first and then increased, while the proportion of low-value areas of coordination level increased and the phenomenon of “polarization” was prominent in individual years, with a trend of two-level differentiation. The nuclear density centers of the coordination level of the PREE system in the eastern, central and western regions all moved to the right during the research period, with the moving trend being roughly the same as the overall one, but with differences in the waveform. The proportion of low-value areas in the eastern region decreased and the difference in the coordination level among provinces and cities was the lowest in 2019; however, the phenomenon of two different polarizations was more serious. The difference in the regional coordination level in central China first decreased and then increased, while the proportion of high-value areas of regional coordination level increased and the coordination level of the regional system tended to further increase, accompanied by the polarization phenomenon. The regional differences in western China were mainly characterized by “high–low” differences. The proportion of low-value areas in the region gradually increased and the difference in the regional coordination level was smaller than the overall difference, but the phenomenon of two-level differentiation was prominent.

Among the influencing factors, per capita GDP, proportion of tertiary industry GDP to total GDP, proportion of environmental pollution control investment to GDP, forest coverage rate, per capita energy production, grain output and the proportion of urban population promoted the coordinated development of the PREE system to varying degrees. In the development of China’s PREE system, attention should be paid to the role of each subsystem within the overall system and the sustainable development of the PREE system should be promoted by optimizing and improving the economic structure, economic benefits, environmental pressure, environmental protection construction, resource utilization, resource conditions, population structure, population quality, etc.

### 7.2. Policy Implications

According to the research results, two policy suggestions are here proposed for the coordinated development of China’s PREE system in the future.

#### 7.2.1. Maintaining and Improving the Coordinated and Stable Development of the PREE System by Optimizing a Single Subsystem and Coordinating Multiple Subsystems

To optimize a single subsystem, a long-term and stable development strategy should be formulated for the population subsystem. In order to achieve this, the way of thinking should be changed from fragmentation, collectivity and responsiveness to integrity, structure and foresight [46]. The population structure should be constantly optimized and the inclusiveness of the birth policy should be enhanced. Other measures could be adopted to deepen the education reform, promote the equalization of basic public education, promote the training of technical and academic talents in different categories and expand the dividend of population quality.

The resource subsystem can be changed from two aspects: resource conditions and resource utilization. Following the principles and policies of giving priority to conservation, protection and natural harmony of natural resources, new renewable energy sources, such as wind energy, solar energy, hydrogen energy and nuclear energy are suggested to be further developed and utilized by adopting modern science and technology. Measures are necessary to optimize the energy system by adjusting the energy consumption structure, replacing the traditional coal resources with renewable energy, innovating the resource utilization efficiency, and strengthening the energy-saving management of key energy-consuming enterprises. Policies are also required to promote the resource utilization, waste recycling and centralized treatment of pollutants.

For the economic subsystem, four characteristics of the wholeness, hierarchy, structure and openness of economic system shall be paid attention to by improving the interaction of economic factors in the economic system and expanding the economic form to realize the consistent development of digital, green and circular economy. Policies shall expand industries such as energy conservation, environmental protection, clean production, clean energy, waste recycling and green service. Measures need to promote the green transformation of industries such as steel, petrochemical and building materials, and promote the electrification of urban public service vehicles. It shall build a market-oriented green technology innovation system, and establish a unified green product standard, certification and marking system. It shall deepen international cooperation in production capacity, build a high-standard free trade area network, and promote the new development pattern of domestic and international dual-cycle with domestic macro-cycle as the main body [47].

For the environmental subsystem, a long-term ecological compensation mechanism should be established. The protection of the environment should be based on natural protection and supplemented by artificial restoration. The balance relationship, self-regulation ability, feedback mechanism, environmental capacity, stability and sensitivity of environmental system should be clearly defined and the interaction among various environmental factors and layers should be handled well. A modern environmental governance system should be established to promote the market-oriented trading of emission rights, energy-using rights, water-using rights and carbon emission rights. Other measures should promote clean heating, industrial kiln treatment, ultra-low emission transformation of non-electric industries, and accelerate the comprehensive treatment of volatile organic compounds and nitrogen oxides emissions. The issue of climate change should be addressed by implementing measures to achieve the goal of peak carbon dioxide emissions and carbon neutrality, and improving the dual control system of total energy consumption and intensity. The system of carbon intensity control should be implemented as the main system and total carbon emission control as the auxiliary one [48], while local and key industries and key enterprises should be supported in taking the lead in reaching the peak of carbon emissions. The safe and efficient use of clean energy should be promoted, the low-carbon transformation of high-carbon emission industries further promoted, and the goal of carbon neutrality achieved on schedule.

In coordinating multiple subsystems, the operation mechanism among systems it should be constantly optimized, the rules of coordinated development among subsystems mastered and the overall sustainable development of a system with high-level, high-efficiency, high-quality and high-standard subsystems promoted; additionally, attention should be paid to optimizing the weak links of a single subsystem and the “cast effect” of system development should be avoided, in order to achieve the collective advancing of each subsystem.

#### 7.2.2. Policies to Address the Issue of Imbalanced Development across Provinces

From a global perspective, a systematic policy control system should be constructed to solve the problem of imbalanced regional coordinated development. According to the development degree of the regional coordinated level, different policies of talent introduction, resource planning, economic development and environmental regulations should be formulated. By strengthening the inter-regional cooperation ability, a reasonable transformation path and management measures should be taken to alleviate the structural contradictions and regional imbalances of the system, so as to improve the sustainable development ability of the system.

From a regional perspective, China’s regional coordinated development strategy during the 14th Five-Year Plan period was implemented in depth, that is, under the overall framework of “great protection, great opening and high quality”, the western region tried to implement the development strategy of “resources determining cities, resources determining production and resources determining people” and realized an efficient flow and an effective allocation of different resource elements across regions with resources as the link [49].

The western regions’ development should be promoted in order to form a new pattern. Northeast China should abandon the traditional way of thinking about industrialization, use modern information technology combined with regional resources, and upgrade the traditional advantageous industries. Those regions should make full use of the economic advantages of state-owned enterprises, lay out large industries, initiate large projects, drive private enterprises with state-owned enterprises and make new breakthroughs in promoting the revitalization of Northeast China.

The central regions should consolidate the ecological green development pattern and realize both development in protection and protection in development. They should actively undertake the layout and transfer of emerging industries, explore the replacement mechanism of developing ecological industries instead of ecological destructive industries, build several high-end green industrial clusters, create a new highland for inland industrial development and create a new situation of rapid rise in central China.

The eastern regions should continue to strengthen the construction of ecological civilization, promote the implementation of an ecological compensation mechanism in the Yangtze River, Yellow River and other important river basins, pay attention to the development and utilization of regional marine resources and build a modern marine industrial system. These regions should encourage the acceleration of modernization, transformation and upgrading, as well as accelerating the gathering of innovation elements, achieving breakthroughs in innovation leading, giving full play to the demonstration role of the eastern regions and actively exporting technical experience to backward areas.

From the provincial perspective, provinces and cities with a low coordination level should determine their own development weaknesses and formulate feasible and effective development plans, while provinces and cities with high coordination level should continue to maintain the coordination and stability of system operation, as well as sharing and leading the development experience. Provinces and cities should re-plan the roles of government, enterprises and the public (individual), together with the performance of powers, duties and obligations. The government should change its role from the identity of “manager” to the dual identity of “manager + server”; business operators should change their roles from that of a traditional rational economic man to that of an ecological rational economic man, while the public (individual) should change its role from “bystander” to “participant”. In addition, the system management mode at different levels and regions and the operation efficiency of the system should be improved.

This study can provide reference for other developing countries to study the coordinated development of the four subsystems of population, resources, economy and environment.

### 7.3. Limitations

This study has certain limitations. Due to the limitation of the data, this study only analyzes the PREE system of 31 provinces and cities in China, which lacks practical significance for the targeted formulation and implementation of regional coordination policies, compared with taking prefecture-level cities and counties as the research object. In addition, when using the spatial error model (SEM) to analyze influencing factors, more influencing indicators should be considered. Therefore, research on the coordinated development of the PREE system should be further expanded from a microscopic point of view.

## Figures and Tables

**Table 2:** Classification of coupling and coordination degree of PREE system. Source: modified based on Table 1 in Chen et al. [35].

Coupling Degree of Coordination	Coupling Coordination Degree D Value Interval	Coupling Degree of Coordination	Coupling Coordination Degree D Value Interval
Extreme imbalance	(0.0–0.1)	Barely coordinated	[0.5–0.6)
Severe imbalance	[0.1–0.2)	Primary coordination	[0.6–0.7)
Moderate imbalance	[0.2–0.3)	Intermediate coordination	[0.7–0.8)
Mild imbalance	[0.3–0.4)	Well coordinated	[0.8–0.9)
On the verge of imbalance	[0.4–0.5)	Quality coordination	[0.9–1.0)

**Table 3:** Mean value of coordination degree of PREE system in various regions of China.

Area	2010	2011	2012	2013	2014	2015	2016	2017	2018	2019
Nationwide	0.382	0.442	0.487	0.542	0.555	0.575	0.602	0.633	0.686	0.718
East	0.400	0.439	0.503	0.536	0.545	0.568	0.597	0.606	0.666	0.708
Central	0.368	0.451	0.494	0.553	0.552	0.568	0.608	0.637	0.660	0.684
West	0.375	0.437	0.467	0.541	0.565	0.588	0.603	0.655	0.721	0.750

Note: According to the classification of the National Bureau of Statistics of China, the eastern region includes Beijing, Tianjin, Hebei, Liaoning, Shanghai, Jiangsu, Zhejiang, Fujian, Shandong, Guangdong and Hainan; the central region includes Shanxi, Jilin, Heilongjiang, Anhui, Jiangxi, Henan, Hubei, Hunan; the western regions include Inner Mongolia, Guangxi, Chongqing, Sichuan, Guizhou, Yunnan, Shaanxi, Tibet, Gansu, Qinghai, Ningxia and Xinjiang.

**Table 4:** LISA clustering results of the coordinated development level of China’s provincial PREE system.

Year	H–H Quadrant	L–L Quadrant	L–H Quadrant	H–L Quadrant
2014	—	Shandong Province (0.05)	Inner Mongolia Autonomous Region (0.05)	Jiangsu Province (0.05)
2017	Gansu province (0.05), Ningxia Hui Autonomous Region (0.05)	Hebei Province (0.05), Tianjin City (0.05)	—	—
2018	Xinjiang Uygur Autonomous Region (0.05), Gansu province (0.01), Tibet Autonomous Region (0.01), Qinghai Province (0.05), Ningxia Hui Autonomous Region (0.05)Sichuan Province (0.05)	Liaoning Province (0.05)	—	—
2019	Xinjiang Uygur Autonomous Region (0.05), Gansu province (0.01), Tibet Autonomous Region (0.01), Qinghai Province (0.05), Ningxia Hui Autonomous Region (0.05)	—	—	—

**Table 5:** Descriptive Statistics of Variables.

Variable	Obs	Mean	Std. Dev	Min	Max
Y (%)	310	0.56	0.11	0.26	0.81
GDP (Yuan/person)	310	51,951.19	26,187.98	13,119.00	164,220.00
TI (%)	310	45.97	9.69	28.60	83.5
EP (%)	310	1.33	0.77	0.06	4.66
FC (%)	310	32.08	17.90	4.02	66.80
EPC (Tons/person)	310	3.68	6.18	0.02	42.73
GY (Tons/ha)	310	4.29	1.70	1.35	8.25
UP (%)	310	56.09	13.39	22.67	89.60
PC (Ten thousand people)	310	83.50	51.97	3.11	231.97

**Table 6:** Statistics of LM test results.

Check Type	LM-Error	LM-Lag	RobustLM-Error	RobustLM-Lag
Statistic (*p*-value)	9.871 (0.002)	0.178 (0.673)	29.219 (0.000)	19.526 (0.000)

**Table 7:** Statistics of Hausman test results.

Test Summary	Chi^2^ (9)	Prob > Chi^2^
Test	28.05	0.0009

**Table 8:** Regression results.

KERRYPNX	OLS	SEM	
		Time-Fixed Effect	Individual-Fixed Effect
GDP	0.2643 ***	−0.1078 ***	0.3968 ***
	(5.62)	(−3.54)	(6.21)
TI	0.4238 ***	−0.1621 ***	0.2708 ***
	(5.48)	(−3.15)	(3.74)
EP	−0.0246	0.0270 **	0.0343 ***
	(−1.27)	(2.23)	(2.59)
FC	0.0662 ***	0.0141	0.2460 ***
	(3.77)	(1.39)	(2.57)
EPC	0.0658 ***	−0.0018	0.1427 ***
	(7.00)	(−0.29)	(6.29)
GY	0.0526	−0.0113	0.1692 ***
	(1.28)	(−0.48)	(3.41)
UP	−0.2591 ***	0.3574 ***	0.7066 ***
	(−3.06)	(6.67)	(3.38)
PC	−0.0217	−0.0341 ***	0.0072
	(−1.16)	(−3.23)	(0.08)
_cons	−4.2353 ***		
	(−14.06)		
λ		−0.0077	0.1353
		(−0.09)	(1.63)
sigma2_e		0.0098 ***	0.0075 ***
		(12.45)	12.42
N	310	310	310
R2	0.3522	0.4335	0.8259
Log-likelihood		276.2759	316.9871

Note: The regression coefficients are the standard errors in parentheses. *** and ** indicate significant at the levels of 1%, and 5%, respectively. The time-fixed effect is to solve the problem of missing variables that do not change with individuals but change with time, while the individual-fixed effect is to solve the problem of missing variables that do not change with time but vary from individual to individual.

## Data Availability

The data presented in this study are available on request from the corresponding author.
